# Cumulative BRCA mutation analysis in the Greek population confirms that homogenous ethnic background facilitates genetic testing

**DOI:** 10.1186/s13053-015-0037-y

**Published:** 2015-08-19

**Authors:** Alexandra Tsigginou, Fotios Vlachopoulos, Iordanis Arzimanoglou, Flora Zagouri, Constantine Dimitrakakis

**Affiliations:** 1 Breast Unit of the 1st Department of Obstetrics and Gynecology, Athens University Medical School, Athens, Greece; 2 Genomedica, S.A, Piraeus, Greece; 3 Department of Clinical Therapeutics, Alexandra Hospital, Athens University Medical School, Athens, Greece

**Keywords:** BRCA, Molecular alteration, Pathogenic mutation, Molecular genetic testing, Family/personal history, Inherited breast cancer, Greece

## Abstract

Screening for BRCA 1 and BRCA 2 mutations has long moved from the research lab to the clinic as a routine clinical genetic testing. BRCA molecular alteration pattern varies among ethnic groups which makes it already a less straightforward process to select the appropriate mutations for routine genetic testing on the basis of known clinical significance.

The present report comprises an in depth literature review of the so far reported BRCA 1 and BRCA 2 molecular alterations in Greek families. Our analysis of Greek cumulative BRCA 1 and 2 molecular data, produced by several independent groups, confirmed that six recurrent deleterious mutations account for almost 60 % and 70 % of all BRCA 1 and 2 and BRCA 1 mutations, respectively.

As a result, it makes more sense to perform BRCA mutation analysis in the clinic in two sequential steps, first conventional analysis for the six most prevalent pathogenic mutations and if none identified, a second step of New Generation Sequencing-based whole genome or whole exome sequencing would follow. Our suggested approach would enable more clinically meaningful, considerably easier and less expensive BRCA analysis in the Greek population which is considered homogenous.

## Introduction

Breast cancer is the most common malignancy among women in Greece involving 23 % of all types of cancers [1]. According to World Health Organization ~87 women in a 100.000 Greek population will develop breast cancer in their life time [2] with a relatively small proportion of it to be classified as hereditary. Multiple genes have been recognized to confer a risk of familial breast cancer, but in more than 70 % of familial breast cancer cases the genetic factor remains unclear. In the remaining proportion, genetic testing can identify a pathogenic mutation of *BRCA 1* or *BRCA 2* in more than 25 % of the affected families while other known genes namely *TP53, PTEN, STK11, CHEK2, ATM* may account for no more than 1 % of the total familial breast cancer [2, 3]. The inheritance of a deleterious mutation in one of the two breast cancer susceptibility genes, *BRCA 1* and *2* , is associated with a high lifetime risk of breast cancer, currently estimated at 65 % (CI 44-78 %) for *BRCA* 1 and 45 % (CI:31-56 %) for *BRCA 2* [3]. Deleterious mutations in *BRCA* genes also increase the lifetime risks of ovarian cancer and predispose to a range of other malignancies.

It is widely accepted that the distinct differences in cancer incidence and mortality observed among different ethnic groups may appear due to ethnic background dependent variant genetic component associated with the disease combined to other non-genetic, epidemiologic and life style connected risk factors. It is also documented that different mutations concerning specific predisposition gene(s), in our case *BRCA 1* and *BRCA 2*, may characterize different ethnic populations. Inherited gene alterations which persistently appear from generation to generation in specific ethnic groups can characterize those groups/populations and constitute what is known as founder effect, which apparently is due to a shared common ancestry [4]. According to the literature, the founder effect is the reduction in genetic variation or a gene mutation observed in high frequency in a specific population (add reference). Thus, it makes sense from the clinical point of view to retrospectively analyze cumulative *BRCA* molecular data for *BRCA* alterations confined to the Greek population, which is considered over 95 % genetically homogeneous. Ultimately, this might help in developing a meaningful, comprehensive and cost-effective *BRCA* diagnostic and prognostic tool to be offered to high-risk individuals or breast/ovarian cancer patients [5].

*BRCA 1* and *BRCA 2* genes normally produce tumor suppressor effect and it is commonly accepted that their germline mutations account for the majority of the identified and diagnosed familial and hereditary breast and ovarian cancers [6, 7]. Breast cancer attributed to *BRCA 1* and *BRCA 2* mutations tend to appear in younger age and exert a more aggressive phenotype [8, 9]. Thus, genetic testing for predisposition to breast cancer before the spring of the disease and a more accurate molecular diagnosis are both essential in applying preventive measures to individuals at risk and, for women with cancer, patient stratification towards a more personalized medical treatment in the long run. In this context, genetic testing for *BRCA 1* and *BRCA 2* is recommended to breast cancer patients diagnosed before age of 50, with bilateral breast cancer, and/or triple negative breast cancer, and/or history of ovarian cancer, and/or more than two first degree relatives with breast or ovarian cancer. Following a positive for mutation diagnosis, other affected and unaffected members of the family are screened for the specific mutation. Genetic testing should also be considered for women with distinct family history of breast and/or ovarian cancer where affected relatives cannot be screened [10, 11].

Despite the extensive *BRCA* mutation screening of Greek patients and individuals at risk, there is not yet clear consensus as to what should be the most clinically appropriate *BRCA 1* and *BRCA 2* mutation panel for the Greek population. The present report constitutes a thorough analysis of the so far identified mutations in Greek families aimed to contribute to the real need to develop a standard and comprehensive *BRCA* routine molecular test in the clinic.

## Methods

We conducted a PubMed literature inquiry using as key words: Greek/breast cancer and genetics, which resulted in 99 studies. We then reviewed all papers reporting *BRCA 1* and/or *BRCA 2* molecular alterations and mutations in the Greek population and we found that only 14 of the total 99 studies were originally designed to solely include Greek families and patients. Cumulative Greek family data were collected and analyzed for the purpose to more precisely correlate, and perhaps associate specific *BRCA 1* and *2* mutations to the Greek ethnic group.

## Results - review

Currently, the exact proportion of breast cancer in Greece with documented genetic component cannot be accurately predicted, but it is believed to be comparable to the European rates. For the *BRCA 1* and *2* genes in particular, which have long gained the attention of the global bio community, a large amount of Greek (resident within Greece or Greek-immigrant families) and Greek-Cypriot data have been produced. Cumulative Greek *BRCA* mutation data are shown in Table 1.

**Table 1:** BRCA 1 and BRCA 2 mutations found in a Greek population. (Mutations repeatedly found in multiple papers share the same font colors)

Author	Pathogenic mutation		Sample/methods used	Results
	BRCA1	BRCA2		
Konstantopoulou et al., 2000 [12]	3741insA, 1623del5-TTAAA		30 breast/ovarian cancer pts with family history	5 mutations & 6 polymorphisms
	5382insC, 3099delT, 3277insG			
			PTT, direct sequencing	
Armakolas et al., 2002 [18]		2024del5 3058delA 6024del TA 4147del G	55 bc pts: 27 with family history,28 sporadic bc	4 different mutations in 5 pts
			SSCP, sequencing	
Ladopoulou et al. 2002 [14]	5382insC non-sense R1751X 5586G > A	2024del5 3034del4 6631del5	85 pts with positive history	Mutations in 14 families (16.5 %)
			PTT, SSCP, direct sequencing	
Kroupis et al., 2003 [13]	5382insC		Study of one affected family,	
Belogianni et al., 2004 [16]	exon 20: 5331G > A, 5382insC , entire exon 20 deletion exon 23:5586 G > A		25 individuals of 18 fms/12 high risk	5 fms were positive for mutation
			dHPLC, MLPA, Long PCR	
Kataki et al., 2005 [19]	2306A > T, 4750C > A, 5129A > C, 5627G > T, IVS8-19delT, 2196G > A, 4793A > G, 4956A > G, 4610 T > C	4147delG (2 pts), 3058delA, 6024delTA, 2024del5, 385A > G, 360 T > G	94 individuals with low or moderate risk to be carriers	BRCA1: 5 unclassified variants & 4 polymorphisms BRCA 2: 5 unclassified variants & 1 polymorphism
			PTT, SSCP , sequencing	
Armaou et al., 2007 [15]	exon 20:71146-75319del, 71618-74863del exon 24:82651-87079del, 82651-87079del		95 pts with positive family history (one case of sporadic Ca)	
			QMPSF, diagnostic PCR primers	
Anagnostopoulos et al., 2008 [20]	5331G > A (G1738R)		287 breast/ovarian cancer families Study specific for G1738R mutation/PCR sequencing	
Konstantopoulou et al., 2008 [21]	exon 20: 5382insC, 5331G > A (G1738R)	3058delA 2024del5	127 bc/ovarian cancer fms	16 fms BRCA1 mut & 5 fms BRCA2 mut
			diagnostic PCR primers, dHPLC	
Armaou et al. 2009 [23]	c.5266dupC, G1738R, and two deletions of exons 20 and 24		987 unselected pts examined for specific mutations	2.6 % carriers
			diagnostic PCR primers, PCR	
Koumpis et al. 2011 [22]	exon 20: 5331G > A, 3.2 kb deletion exon 11: 3819delGTAAA	exon 11: 3782del10, 4512insT	127 unselected sporadic bc patients	6 carriers found (no family history)
			diagnostic PCR primers, PCR, ABI, PTT	
Pertesi et al. 2011 [24]	exon 20:del D17S579 - D17S1299 (3.9 Mb) exon 24: del D17S951 , D17S1299 (2.9 Mb)		Study of affected families	
			diagnostic PCR primers (10 short tandem repeat markers)	
Fostira et al., 2014 [25]	exon 11- (c.3178G > T)		Case report of a carrier with negative family history	
Konstantopoulou et al. 2014 [26]	exon 5: (300 T > G - C61G) exon 7: (449delG-ter118)* exon 11: (1329insCT)*, (1623del5), (1624 T > G - L502X)*, (1806C > T - Q563X)*, (2072del4), (2767insGGCA)*, (3082C > A - S988X)* ,(3297G > T - E1060X), (3494delTC), (3726C > T - R1203X), (3741delA)*, (3819del5), (3874del4) exon 12: (4286delTG)* exon 14: (4510delCTAinsTT) exon20: (5331G > A - G1738R), (5370C > T - R1751X), (5382insC), (g.71660_74860del3200) exon 21: (5447delC) exon 22: (IVS22 + 5G > C) exon 23: (5550C > T -Q1811X)*, (5586G > A), (g.80280_91331del11052) exon24: (5611delC), (g.82651_87079del4429_ins5)	exon8- (886delGT) exon 11: (2567C > G - S780X), (3036del4), (4643del4), (4997delA), (5950delCT), (6718C > T - Q2164X), (6828delTT) exon 17: (IVS16-2A > T)*, (8204G > A) exon 19: (8592G > A - W2788X) exon 22: (9158delA) exon23: (9218del32) exon 24: 9325insA exon 25: 9604C > T - Q3126X) exon 27: (9976insT)	473 breast/ovarian cancer patients with family history	32 % mutation prevalence. 44 mutations found (6 BRCA1 recurrent/founder mutations dominate the observed spectrum-58.5 % of all mutations found)
			diagnostic PCR primers, direct sequencing , MLPA	

(*pts* patients, *bc* breast cancer, *fms* families, *mut* mutation)

*PTT* Protein truncation test

*SCCP* Single-Strand Conformation Polymorphism Analysis

*dHPLC* denaturing High Performance Liquid Chromatography

*MLPA* Multiplex Ligation – dependent PCR Amplification

*QMPSF* Quantitative Multiplex PCR of Short Fluorescent Fragments

*ABI*, TaqMan Copy Number Variation Assays

*: novel mutations (the time the study was published) (footnote), indication deleted on the last cell of the table

Historically, the first reported study in a Greek population by Konstantopoulou et al. (appeared in the literature in 2000) examined for *BRCA 1* mutations 30 breast/ ovarian cancer patients with strong or moderate family history and premenopausal age at onset of the disease [12]. Three mutations previously described in non-Greek populations, (*3741insA*, *1623del5*-*TTAAA*, *5382insC*) and two novel ones (*3099delT*, *3277insG*) were found. This finding was the first evidence that *BRCA* 1 mutations may account for as much as 20 % of high-risk Greek families, incidence comparable to other European countries. This was also the first report correlating two novel mutations to the Greek population. Later, in 2003, Kroupis et al. reported a family affected with atypical medullary breast carcinoma carrying the *5382insC* mutation and thus confirmed that this rather universal *BRCA 1* mutation is detected in Greece too [13]. Later, the exact same mutation was found to be the most frequent mutation in Greek breast/ovarian cancer families [14]. Ladopoulou et al. examined eighty five patients with positive family history for *BRCA 1* and *BRCA 2* deleterious mutations and 14 were found to carry 6 mutations: the aforementioned *5382insC*, the non-sense *R1751X* and the *5586G > A* of *BRCA* 1, and frameshifts *2024del5*, *3034del4*, and *6631del5* of *BRCA 2*. Molecular alterations of yet unknown biological significance including some with higher than 1 % frequency and thus potentially constituting polymorphisms were also reported.

Armaou et al. studied 95 patients of Northern Greece, for *BRCA* 1 molecular alterations. Four deleterious deletions in exons 20 and 24 were found in this study, one of which was reported to be novel. There were two carriers of that newly identified mutation; one developed breast cancer at 28 years, ovarian cancer at 31 and endometrioid cancer at 38 and the second carrier developed ovarian cancer at 52 and breast cancer at 53 years. Hence, this mutation is a strong candidate to be included in the Greek *BRCA 1* mutation panel to diagnose women with high risk to develop early onset breast and ovarian cancer [15].

In another study, limited in Greece and Cyprus, 18 affected families examined for *BRCA 1* mutations and four different mutations were found in five families : Mutation 5382insC in exon 20, missense mutation *5331G > A* in exon 20 , single base substitution G > A at nucleotide *5586* in exon 23 and a first reported deletion of the entire exon 20 [16]. Also, it is worth mentioning that these mutations were associated to early onset breast cancer (occurring in 29 to 50 years) with variable penetration. Ovarian cancer presented in only one patient and there were also cases of colorectal, larynx and lungs neoplasms within these families, a pattern that resembles to Li Fraumeni cancer syndrome. The deletion of the entire exon 20 was identified in one Greek family and in one Italian family generated from different DNA alterations. Two unaffected mutation carriers aged 39 and 55 were diagnosed, suggesting that calculating genetic risk based on *BRCA 1* alone may in fact be less informative than we currently think [17].

In 2002, the first study with Greek *BRCA 2* mutations in breast cancer patients was published [18]. Armakolas et al. examined five distinct breast cancer families, and found one carrying the known germline deleterious mutation (*2024del5*) and three other families carrying novel mutations (*3058delA*, *6024delTA*, and *4147delG*). It is noteworthy that breast cancer patients with tumors carrying these mutations exhibited more favorable clinical phenotype compared to stage matched sporadic cases, irrespective of histological type. Also, it was observed that in a Greek population, this more favorable phenotype was correlated to a cluster of novel mutations in exons 10 and 11 [18].

Following, Kataki et al. examined 94 individuals with low or moderate risk for *BRCA 1* and *2* mutations based purely on family history [19]. Three novel *BRCA1* missense mutations, one novel *BRCA 1* intronic deletion, three *BRCA 2* truncating mutations, and one novel *BRCA 2* missense mutation were identified.

According to the results of Anagnostopoulos et al. study, which involved 11 Greek families residents of Greece, Australia and USA, one missense mutation at *5331G > A* of exon 20, accounts for 3 % and 12.3 % of all breast/ovarian cancer families and all carriers of deleterious mutations, respectively [20]. These results coupled to genealogical history, point to *5331G > A* mutation as a likely Greek founder effect. Not surprisingly, our analysis confirmed that the most common pathogenic *BRCA 1* mutation in the Greek population is the *5382insC* located also in exon 20, widely known as the Jewish founder, which remains to be the most common (10 % of all *BRCA 1* mutations) alteration amongst Caucasians worldwide, and therefore, can be associated with the Caucasian race [21]. Alternatively, our analysis further supports the view that *5382insC* is not an ethnic-specific characteristic but rather a “universal” Caucasian mutation. Unlike *5382insC*, evidence indicates that the *BRCA 2* mutations subst *G > A 5586* in exon 23 for *BRCA 1* and *2024del5* and *3058delA* can be considered as Greek-specific mutations [21].

Koumpis et al. [22] examined unselected by family history, breast cancer patients and six mutations were found (four in *BRCA 1* and two in *BRCA 2*) which account for 4.7 % of the total and for 9.5 % of cases diagnosed before age forty. This result that breast cancer incidence doubles before the age of forty adds to the current belief of strong genetic component of early breast cancer onset. Recently, we have also published a series of breast cancer in women aged 25 years and younger where, the percentage of BRCA mutations raised to 25 % of cases in this very young age group. The overall incidence of BRCA mutations found in Koumpis study is twice that found by Armaou et al. who studied only four selected mutations in Greek women with breast cancer [23] but in line with results published by others having studied non Greek ethnic background women. Notably, Koumpis et al. reported that only one of the six mutation carriers reported family history of breast or ovarian cancer, which strongly favors the current belief that many carriers share no previous family history of the disease. Taken together, the above data clearly indicate both, that other than the aforementioned *BRCA* mutations play a role in inherited Greek breast cancer and that breast cancer susceptibility may involve other than the *BRCA* genes, consistent with the results published by International breast and ovarian cancer genetic predisposition consortia (http://www.ncbi.nlm.nih.gov/pubmed/25581431 and http://www.ncbi.nlm.nih.gov/pubmed/25452441.

Before us, Koumpis et al. supported the view that mutation *5331G > A* of exon 20 is likely to be founder effect in the Greek population [22]. In the same study, two novel *BRCA 1* mutations, one 3.2 kb deletion in exon 20 and one in exon 11 (*3819delGTAAA*) and two *BRCA 2* mutations in exon 11 (*3782del10*, *4512insT*), were found. In addition, Pertesi et al. supported the view that the deletions of 3.9 Mb of exon 20 and 2.9 Mb of exon 24 may be Greek ethnic group-associated molecular events [24]. Furthermore, Fostira et all described the *p.E1060X* aggressive mutation in *BRCA 1*/exon 11 (c.*3178G* > T) that affected a young woman with no family history. The inheritance proved to originate from the paternal family that counted very few female members, fact which highlights the necessity of genetic testing even in cases with negative family history [25].

In a recent study, Konstantopoulou et al. [26] conducted a large survey screening 473 Greek breast/ovarian cancer families for *BRCA 1* and *BRCA 2* mutations. This study resulted in 28 different deleterious *BRCA 1* mutations (9 novels, firstly identified) and 16 deleterious BRCA 2 mutations (7 novels) which apparently set the basis for the development of a diagnostic tool applicable to diagnose Greek familial breast/ovarian cancer. Interestingly, the great majority (up to 75 %) of the *BRCA 1* families were positive for mutations located at the 3’end of the gene that include exons 20 to 24. Alternatively, almost 60 % of all Greek familial breast/ovarian cancer appear to be attributable to *BRCA 1* mutations in the same region of the gene which, understandably, can streamline the whole mutation analysis process.

It has been now two decades of intense research targeted to *BRCA 1* and *2* mutations covering a wide range of different ethnic populations. Most of the Greek studies cited here have predominantly focused on examining families with positive history in accordance with the current guidelines which recommend focusing *BRCA* genetic screening on unaffected members of affected families [27]. We now know that this biased approach towards breast cancer cases selected on a family history basis, has provided limited information as per the actual prevalence of *BRCA 1* and *2* mutations in the Greek population and the actual penetrance of the disease in carriers. Moreover, increasing evidence indicates that a large fraction of families with a strong family history of breast cancer scores negative for *BRCA* mutations which underlines the importance to always make a clear distinction between familial and inherited breast cancer, the former being a small subset of the latter. New genes, like *PALB 2*, are likely to emerge as the next breast cancer susceptibility gene(s) and their screening will gradually intensify, beyond any doubt [8]. However, this accumulative data confirms that in a homogenous ethnic population like Greek, common *BRCA* mutations can facilitate genetic testing.

## Conclusions

Considerable effort has been made to detect and more accurately predict the *BRCA 1* and *2* mutations with the highest frequency in the homogeneous Greek population. Our analysis of cumulative Greek *BRCA* data clearly indicates that six specific mutations account for almost 60 % of *BRCA 1* and *2* mutations and 70 % of *BRCA 1* mutations. This observation strongly supports the view that a two-step BRCA analysis procedure might be more meaningful to be implemented in the Greek clinic. In other words, in clinical routine, every Greek woman referred for *BRCA* screening can undergo screening for the six known mutation first and if the first step fails to diagnose a deleterious mutation, then a comprehensive New Generation Sequencing-based whole genome or whole exon analysis, as previously described by others (indicatively, http://www.ncbi.nlm.nih.gov/pubmed/25859162 and http://www.ncbi.nlm.nih.gov/pubmed/25896959) will follow. Only after *BRCA* genetic testing of tens of thousands of Greek women will have been completed, we will be in a position to more accurately evaluate whether the suggested six mutation panel is the appropriate one or needs to be extended and/or changed for the purpose of the daily routine *BRCA* testing in the clinic and whether one or more of these or new mutations may constitute a Greek founder effect.
